# Matching the Optimal Operating Mode of Polydimethylsiloxane Check Valves by Tuning the Resonant Frequency of the Resonator in a Piezoelectric Pump for Improved Output Performance

**DOI:** 10.3390/mi14010015

**Published:** 2022-12-21

**Authors:** Jian Chen, Fanci Meng, Zihan Feng, Wenzhi Gao, Changhai Liu, Yishan Zeng

**Affiliations:** School of Mechanical Engineering, Hefei University of Technology, Hefei 230009, China

**Keywords:** piezoelectric pump, PDMS check valve, side-curling mode, resonant frequency matching

## Abstract

This paper proposes to improve the output performance of a piezoelectric pump by matching the resonant frequency of the resonator to the optimal operating mode of bridge-type polydimethylsiloxane (PDMS) check valves. Simulation analyses reveal that the side-curling mode of the PDMS valve is conducive to liquid flow and exhibits a faster frequency response compared with the first bending mode. The first bending resonant frequency of a beam-type piezoelectric resonator was tuned close to the side-curling mode of the PDMS valve by adjusting the weight of two mass blocks installed on both ends of the resonator, so that both the resonator and the valve could work at their best conditions. Experiments were conducted on a detachable prototype piezoelectric pump using PDMS valves with three different lengths. The results confirm that the peak flowrate at the resonant point of the pump reaches its maximum when the resonant frequencies between the resonator and the valve are matched. Maximum peak flowrates of 88 mL/min, 72 mL/min and 70 mL/min were achieved at 722 Hz, 761 Hz and 789 Hz, respectively, for diaphragm pumps using five-, four- and three-inlet-hole PDMS valves, under a driving voltage of 300 V_pp_.

## 1. Introduction

Micropumps have undergone significant development in recent years because of their important and diverse applications in health and engineering [1,2]. According to the physical mechanism, micropumps can be divided into non-mechanical and mechanical micropumps. Non-mechanical micropumps convert non-mechanical energies to kinetic momentum for fluid pumping, and the driving mechanisms include magnetohydrodynamic (MHD) [3], electrohydrodynamic (EHD) [4,5], electroosmotic (EO) [6], etc. Mechanical micropumps utilize physical actuators to perform fluid pumping, and the driving mechanisms include piezoelectric [7], electromagnetic (EM) [8], electrostatic [9], etc.

Piezoelectric pump is a type of mechanical pump with piezoelectric vibrator as the power element, in which electric energy is converted into mechanical vibration through inverse piezoelectric effect, and the mechanical vibration is transformed into unidirectional liquid flow typically by a diaphragm pump [2,7]. Since piezoelectric pumps have many advantages such as a compact structure, good reliability, low power consumption, high control accuracy, and no electromagnetic interference, they have shown broad application prospects in liquid cooling systems [10,11], drug delivery [12,13], robotics [14] and biomedicine [15,16].

According to the working modes of the piezoelectric vibrator, piezoelectric pumps can be divided into two categories: non-resonant type and resonant type. In non-resonant piezoelectric pumps, the piezoelectric element is generally bonded directly to the pump chamber diaphragm, such as the circular piezoelectric driver designed by Liu [17]. The chamber deformation is restricted due to the quasi-static working mode of the piezoelectric vibrator; thus, the output performance of non-resonant piezoelectric pumps is limited. In resonant piezoelectric pumps, the piezoelectric vibrator is usually separated from the pump chamber diaphragm, and different resonant modes are utilized to oscillate the pump chamber with amplified vibrating amplitudes [18,19]. Since the piezoelectric vibrator works at the resonant state, resonant piezoelectric pumps can transmit fluid at large flowrates and backpressures, such as the folded piezoelectric vibrator proposed by Wang [20], and the U-shaped piezoelectric resonator designed by Chen [21].

According to different flow guiding elements, piezoelectric pumps can also be divided into two categories: valveless type [22,23] and valve type [24,25]. Although valveless piezoelectric pumps possess simple structures and better high-frequency characteristics, their power and efficiency are poorer than pumps with valves [26]. The valves used in piezoelectric pumps can be classified into active valves [27] and passive valves [28]. Active valves require an additional control unit and power consumption, while passive valves operate by the pressure difference across the valve. The output performances of piezoelectric pumps using passive valves are highly dependent on the design of the valves, and various types of passive valves have been proposed, such as the cantilever valve [29,30], umbrella valve [31], ball valve [32], and bridge valve [20,33].

Many researchers have used the bridge-type polydimethylsiloxane (PDMS) valves in piezoelectric pumps to achieve high output flowrates, because the lightweight flexible PDMS film opens easily and seals better [34,35]; however, the flat structure of the PDMS valve will deform into a parachute shaped pocket when the valve opens, which produces a large fluid resistance to the flowing liquid [36]. Ye added a blocking edge over the center line of the valve film to modify the deformation pattern of the PDMS valve; as a result, the performance of the PDMS valve was improved dramatically [37].

Inspired by Ye’s work, the work reported in this paper inferred that the first bending mode of the PDMS valve was not conducive to liquid flow, and modal simulations were conducted to seek a more suitable operating mode of the PDMS valve. It was found that the deformation pattern of the PDMS valve in the side-curling mode was prone to liquid flow and possessed a faster frequency response, which could be regarded as the optimal operating mode of the PDMS valve. In order to utilize the valve’s side-curling mode in a resonant piezoelectric pump, the first bending frequency of a beam-type piezoelectric resonator was tuned close to the side-curling mode of the PDMS valve by adjusting the weight of two mass blocks installed on both ends of the resonator. The experiments verified that the peak flowrate at the resonant point of the piezoelectric pump reached its maximum when the resonant frequencies between the resonator and the valve were matched to ensure both the valve and the resonator operated at their best conditions.

## 2. Mechanism of the Check Valve and Resonator

### 2.1. Analysis of the Optimal Operating Mode of the Check Valve

**Figure 1:**
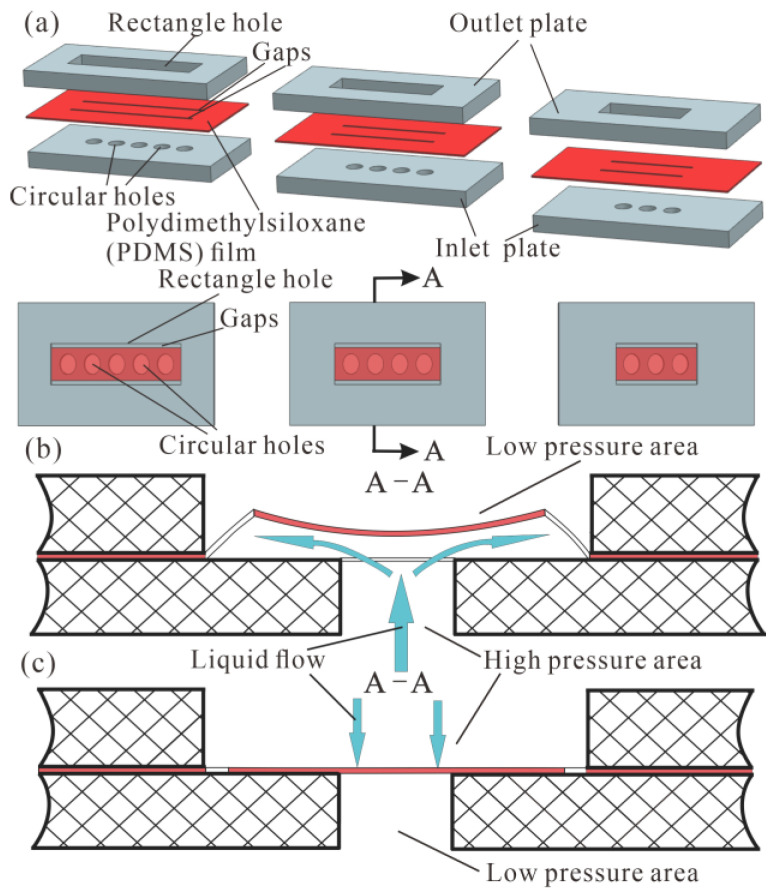
The check valve unit: (**a**) structure of the valve with 5, 4 and 3 inlet holes; (**b**) open mode; (**c**) closed mode.

The check valve unit used in this paper is presented in Figure 1a, which was made of a thin PDMS flexible film sandwiched between an inlet plate and an outlet plate. Several circular holes were engraved on the center line of the inlet plate, a rectangular hole larger than the area of the circular holes was engraved on the center of the outlet plate, and two narrow gaps were cut on the PDMS film along the long edges of the rectangular hole, constructing a bridge valve with two fixed ends. When the pressure at the inlet plate side was higher, liquid flow from the circular holes could push the bridge valve to bend and flow through the rectangle hole to the outlet plate side, as shown in Figure 1b. Otherwise, when the pressure at the outlet plate side was higher, liquid flow from the rectangle hole would push the bridge valve to cover the circular holes and the liquid flow was blocked by the valve from flowing back to the inlet plate side, as shown in Figure 1c.

**Figure 2:**
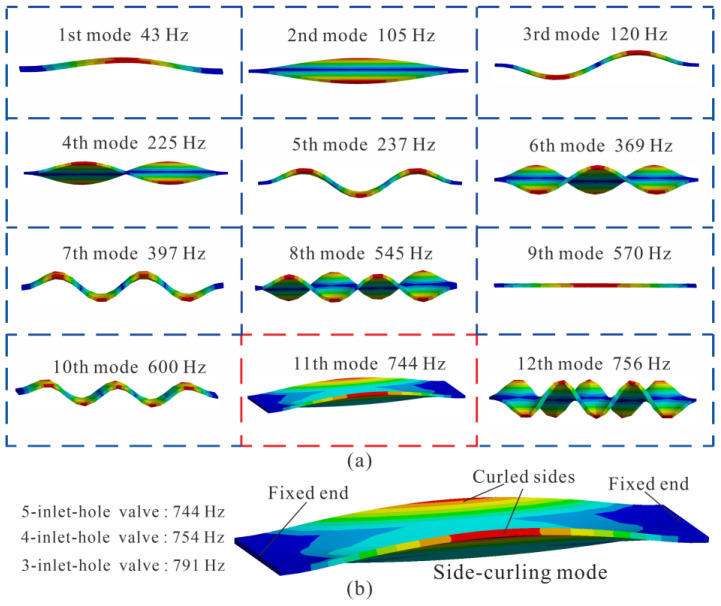
(**a**) The first 12 wet modes of the five-inlet-hole polydimethylsiloxane (PDMS) valve; (**b**) enlarged diagram of the side-curling mode.

In order to verify the repeatability of this study, PDMS bridge valves with three different lengths (shown in Figure 1a) were selected as the check valves in the diaphragm pump. The PDMS valves of different lengths were designed with different numbers of inlet circular holes, so as to maximize the flow cross-sectional area and strengthen the PDMS film when blocking the backflow. The vibration modes of the valves were obtained by applying wet modal simulations in the finite element analysis software, ANSYS 18.0 (ANSYS, Inc., Canonsburg, PA, USA). A PDMS bridge valve was modeled as a flexible beam with both ends fixed and vibrating in liquid water. The thickness of the valves was 0.1 mm, the width was 2 mm, and the lengths were 8 mm, 6.5 mm and 5 mm for the five-, four- and three-inlet-hole valves, respectively. The material properties of the PDMS are listed in Table 1. The first 12 wet modes of the five-inlet-hole valve were obtained as an example for demonstration in Figure 2a, in which the fluid is hidden for better views of the valve vibration modes.

It can be seen from Figure 2a that the promising operating modes of the five-inlet-hole valve were the 1st mode (first bending mode, at 43 Hz) and the 11th mode (side-curling mode, at 744 Hz). In the first bending mode, the valve film deformed into a parachute shaped pocket, which not only produced a large fluid resistance to the flowing liquid, but also forced the liquid confined in the pocket flowing back to the inlet side when the valve film returned to its equilibrium position [36]. By contrast, in the side-curling mode, the valve film deformed along its long edges, which not only was more conducive for the liquid to flow through the valve [37], but also had a faster frequency response. Thus, the side-curling mode can be regarded as the optimal operating mode of the PDMS bridge valve, and the resonant frequencies of the side-curling mode for the five-, four- and three-inlet-hole valves were 744, 754 and 791 Hz, respectively, as shown in Figure 2b.

**Figure 3:**
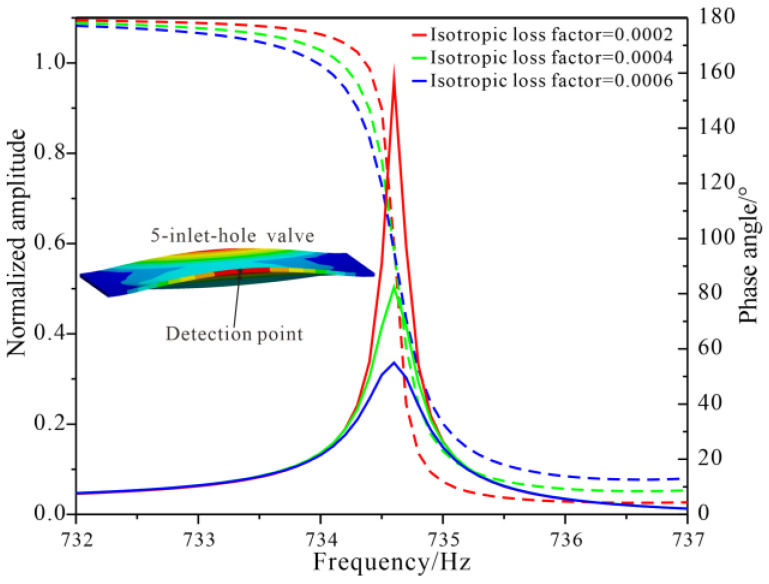
Frequency responses of the five-inlet-hole PDMS valve by harmonic analysis.

The frequency responses of the five-inlet-hole PDMS valve were evaluated using harmonic analyses. In the simulation, liquid water was not modeled for simplification, which led the side-curling mode frequency to be 734.6 Hz; instead, a pressure of 0.1 Pa was applied on the surface of the valve with a frequency range from 732 Hz to 737 Hz in steps of 0.1 Hz, and an isotropic loss factor was employed to simulate the damping effect of liquid water. The frequency responses of normalized amplitude along the vertical direction and phase angle between the vibration of the valve and the driving force were obtained as shown in Figure 3. It can be seen that when the exciting frequency was 734.6 Hz, the amplitude of the valve reached the peak value, at which the phase angle was around 90°; when the exciting frequency was lower than 734.6 Hz, the phase angle was close to 180°; when the exciting frequency was higher than 734.6 Hz, the phase angle was close to 0°; and the larger the damping, the smaller the amplitude peak, and the smoother the phase angle transition from 180° to 0°.

### 2.2. Analysis of the Resonator with Tunable Resonant Frequencies

**Figure 4:**
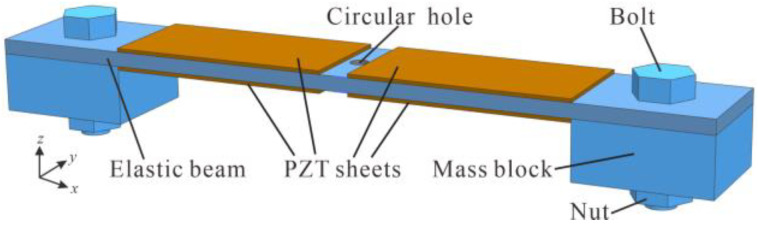
Piezoelectric resonator with tunable resonant frequencies.

The piezoelectric resonator, shown in Figure 4, was composed of an elastic beam, four piezoelectric sheets and a pair of mass blocks. The first bending vibration mode of the resonator was utilized as the working mode to oscillate the diaphragm pump for high pumping performances. In order to coordinate the resonant frequencies between the piezoelectric resonator and the check valve, the first bending resonant frequency of the resonator could be tuned close to the optimal operating frequency of the valve by adjusting the weight of the mass blocks, so that both the resonator and the valve could work at their best conditions.

Both theoretical and simulation analyses were carried out to calculate the resonant frequency of the resonator. Firstly, the resonator was simplified as a Bernoulli–Euler beam without the piezoelectric sheets and mass blocks, and its first bending resonant frequency in free vibration can be expressed as:

(1) $$f_{0}=\frac{4.73^{2}}{2\pi }\sqrt{\frac{E_{1}I}{\rho_{1}Al^{4}}}$$

where *E*_1_, *ρ*_1_, *A*, *l* are the Young’s modulus, density, cross-sectional area, and length of the beam, respectively, and $I=wt^{3}/12$ is the cross-section moment of inertia with *w* and *t* representing the width and thickness of the beam, respectively. Using the parameters listed in Table 1, the first bending resonant frequency of the elastic beam was calculated to be 1063 Hz.

**Figure 5:**
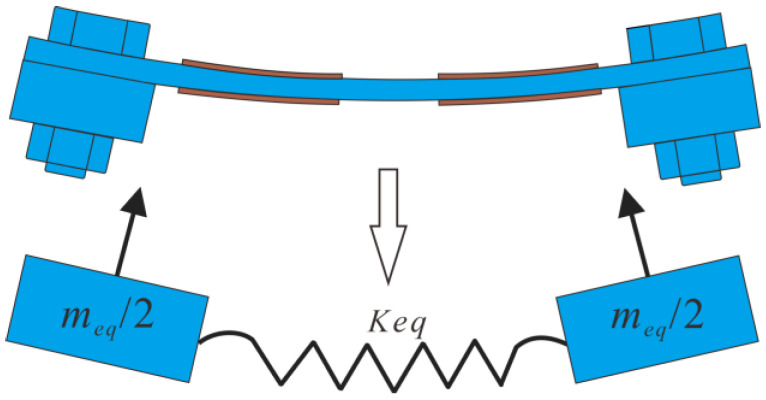
Equivalent model of the resonator with mass blocks.

Secondly, two mass blocks were added to both ends of the elastic beam, and the resonator could be approximately equivalent to a spring mass system, as demonstrated in Figure 5. Thus, the first bending resonant frequency of the resonator can be estimated by the natural frequency of the spring mass system as:

(2) $$f_{1}=\frac{1}{2\pi }\sqrt{\frac{k_{\mathrm{eq}}}{m_{\mathrm{eq}}}}$$

where the equivalent mass *m*_eq_ includes the mass of the beam *m*_0_ and both the mass blocks *m*_1_, and the equivalent stiffness *k*_eq_ is calculated to be 1.03 × 10^5^ N/m when no mass blocks are added (*m*_1_ = 0) and *f*_1_ = *f*_0_ = 1063 Hz. It is assumed that the equivalent stiffness remains unchanged with the adjustment of the mass blocks.

**Figure 6:**
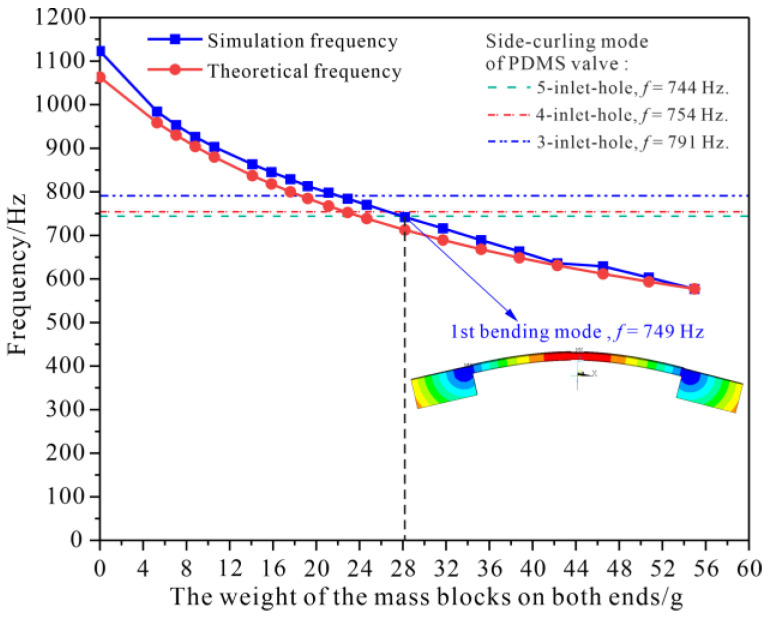
Tuning the first bending resonant frequency of the piezoelectric resonator by adjusting the weight of the mass blocks.

In addition, modal simulations were conducted in ANSYS 18.0 to obtain the relationship between the first bending resonant frequency of the resonator and the weight of the mass blocks. Both the simulation and theoretical results revealed that the resonant frequency decreased approximately inversely with the square root of the weight of the mass blocks, as shown in Figure 6. The tuning range of the first bending resonant frequency was 577~1063 Hz, theoretically, and 577~1123 Hz, in simulation, with the mass blocks’ weight of 0~56 g, which ensured that the resonant frequency of the resonator could be tuned to match with the optimal operating frequencies of the five-, four- and three-inlet-hole valves discussed in Section 2.1.

**Figure 7:**
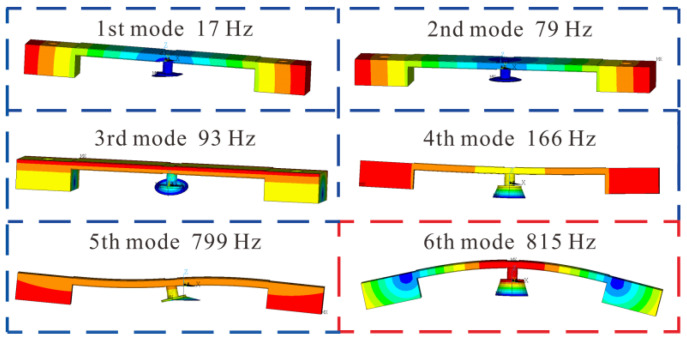
Actuating modes of the piezoelectric resonator with the pump chamber diaphragm.

To evaluate the actual actuating state, the pump chamber diaphragm was attached to the center of the piezoelectric resonator through a linker. A modal simulation in ANSYS was executed with a fixed constraint applied on the circumference of the diaphragm and the mass blocks’ weight of 28.2 g. The first bending resonant mode of the piezoelectric resonator appeared as the 6th vibration mode when actuating the pump chamber diaphragm, as shown in Figure 7, and the resonant frequency of 815 Hz was slightly higher than that of 749 Hz (in Figure 6) under a free vibration with the same mass blocks.

## 3. Design and Fabrication of the Piezoelectric Pump

### 3.1. Design

**Figure 8:**
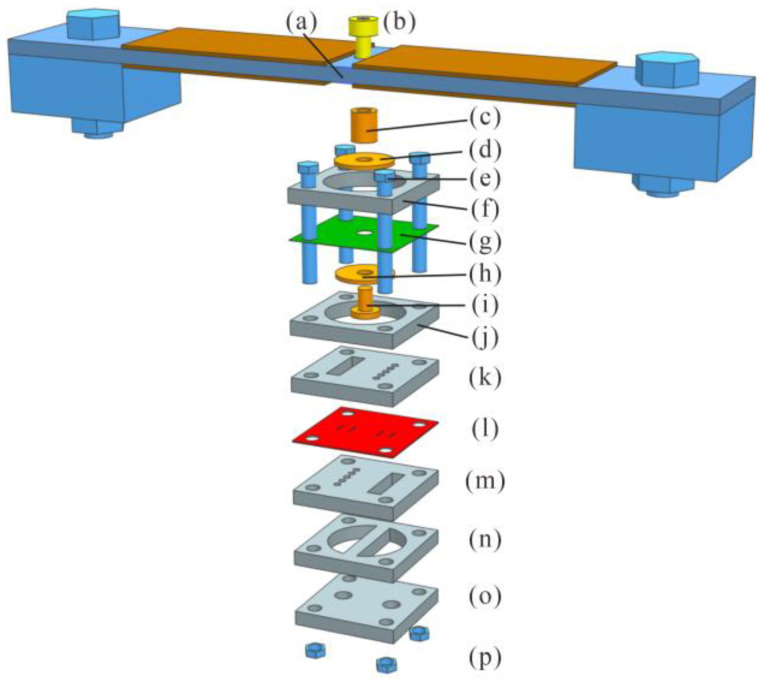
Exploded view of the designed piezoelectric pump: (**a**) piezoelectric resonator; (**b**) screw; (**c**) linker; (**d**) annular gasket; (**e**) bolts; (**f**) polymethylmethacrylate (PMMA) chamber fix plate; (**g**) Kapton diaphragm; (**h**) annular gasket; (**i**) screw; (**j**) PMMA pump chamber plate; (**k**) PMMA outlet/inlet valve seat plate; (**l**) PDMS film of check valves; (**m**) PMMA inlet/outlet valve seat plate; (**n**) PMMA flow channel plate; (**o**) PMMA inlet/out seat plate; (**p**) nuts.

The designed piezoelectric pump included the piezoelectric resonator (detailed in Section 2.2) and a detachable diaphragm pump, as illustrated in its exploded view in Figure 8. The detachable diaphragm pump adopted a laminated structure and consisted of three modules: the pump chamber (Figure 8f,g,j), the check valves (Figure 8k–m) and the flow channels (Figure 8n,o).

The outer surface of the pump chamber diaphragm (Figure 8g) was fixed by two polymethylmethacrylate (PMMA) plates (Figure 8f,j), and the inner surface was stiffened by two annular gaskets (Figure 8d,h). A screw (Figure 8i) penetrated the pump diaphragm through the inner hole of the gaskets to connect with the linker (Figure 8c), which was also connected to the center hole on the piezoelectric resonator (Figure 8a) by another screw (Figure 8b). The check valve module was composed of two valve seat plates (Figure 8k,m) clamping a valve film (Figure 8l), forming an inlet valve and an outlet valve (detailed in Section 2.1). The inlet and outlet (Figure 8o) were connected to the inlet valve and outlet valve (Figure 8m) separately via a flow channel plate (Figure 8n). The three modules of the diaphragm pump were bolted in series by four pairs of bolts and nuts (Figure 8e,p) at the corners of the components, which enabled the diaphragm pump to be detachable and convenient for assembling, disassembling and replacing one or more functional modules.

### 3.2. Working Principle

**Figure 9:**
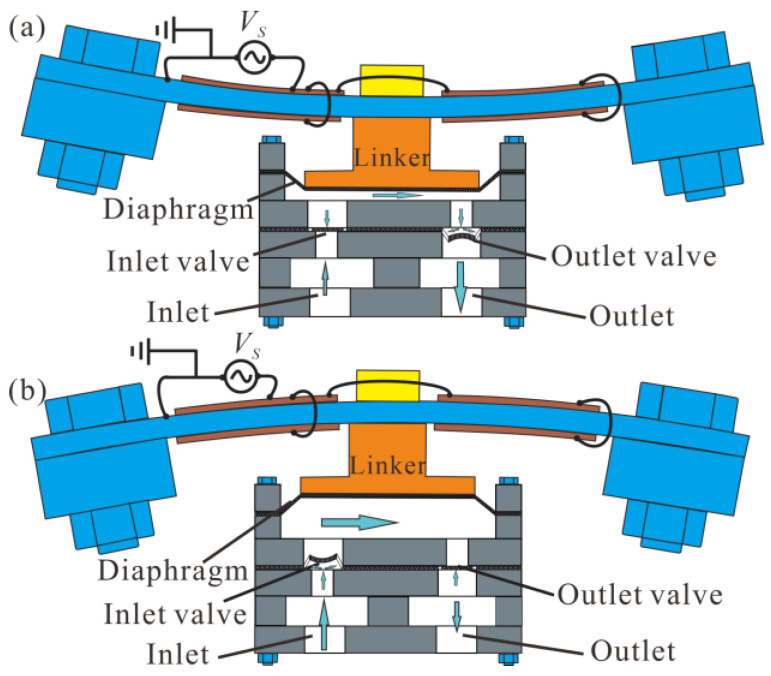
Working principle of the piezoelectric pump: (**a**) dispensing mode, and (**b**) absorbing mode.

The working principle of the piezoelectric pump is presented in Figure 9, which can be generally divided into the dispensing mode (Figure 9a) and absorbing mode (Figure 9b). When the resonator pushes the chamber diaphragm to reduce the chamber volume, the pressure inside the chamber rises. As a result, the inlet valve closes, the outlet valve opens, and the liquid is squeezed out of the pump chamber through the outlet valve (Figure 9a). Alternately, when the resonator pulls the chamber diaphragm to increase the chamber volume, the pressure inside the chamber drops. Consequently, the inlet valve opens, the outlet valve closes, and the liquid is sucked into the pump chamber through the inlet valve (Figure 9b). The dispensing and absorbing modes repeat in turn with the vibration of the resonator, and liquid can be transported from the inlet to the outlet continuously.

In the working process of the piezoelectric pump, electric energy is converted into mechanical vibrating energy by the piezoelectric resonator, the mechanical vibration is transformed into pressure fluctuating in the pump chamber, and the fluctuating pressure is turned into a unidirectional liquid flow by the check valves. If both the resonator and the check valves can work at their optimal conditions at a certain frequency, the liquid transfer capacity of the piezoelectric pump will be improved greatly.

### 3.3. Fabrication

An elastic beam (100 mm × 15 mm × 2 mm) was machined with three circular holes, one in the center and two near both ends. Four PZT-4 piezoelectric sheets (30 mm× 15 mm × 0.2 mm) were adhered to the elastic beam on the surfaces between the three holes using a high-strength epoxy resin glue (DP460). Stainless steel square gaskets were used as mass blocks and bolted to the elastic beam through the two circular holes near both ends.

**Figure 10:**
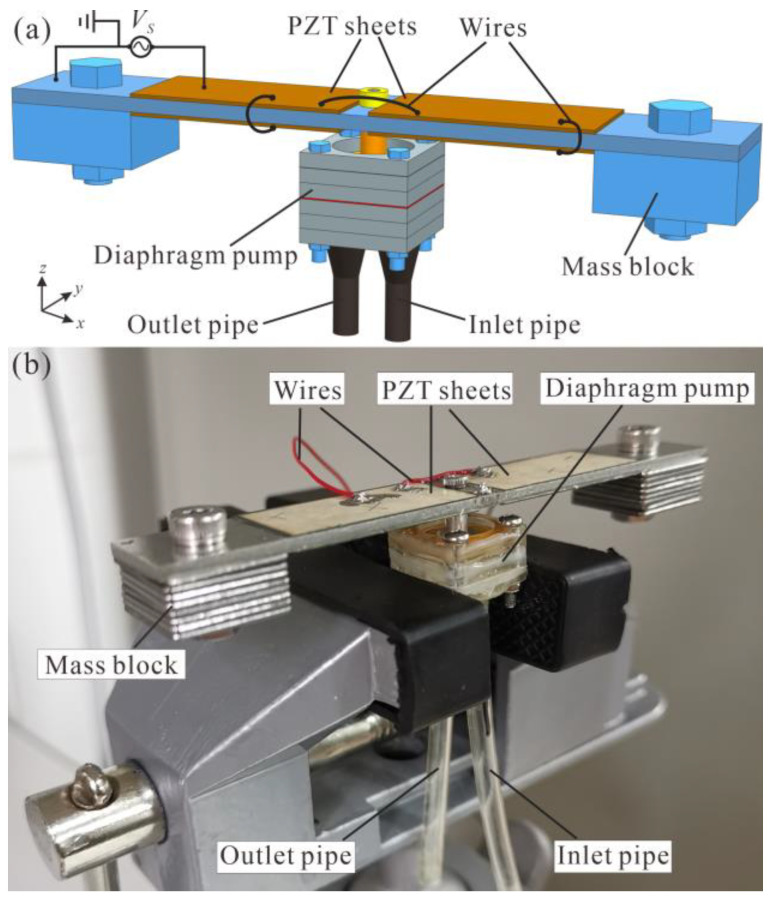
(**a**) Mechanical structure and (**b**) a prototype of the proposed piezoelectric pump.

The three modules in the diaphragm pump were manufactured from six PMMA plates (15 mm × 15 mm × 2 mm), a Kapton film and a PDMS film. The patterns on the PMMA plates were cut by CNC laser processing. The components in every module were bonded by acrylic adhesive for sealing and reinforced by an epoxy resin glue (DP460). A diaphragm pump was assembled by bolts and nuts through the holes at the corners. This construction allowed for interchanging the three types of check valves (using five-, four- or three-inlet-hole valves as described in Figure 1a) without varying the pump chamber module and the flow channel module. At last, the diaphragm pump was bolted to the central hole of the resonator through a screw and the linker, as exhibited in Figure 10.

## 4. Experimental Setup and Results

### 4.1. Experimental Setup

**Figure 11:**
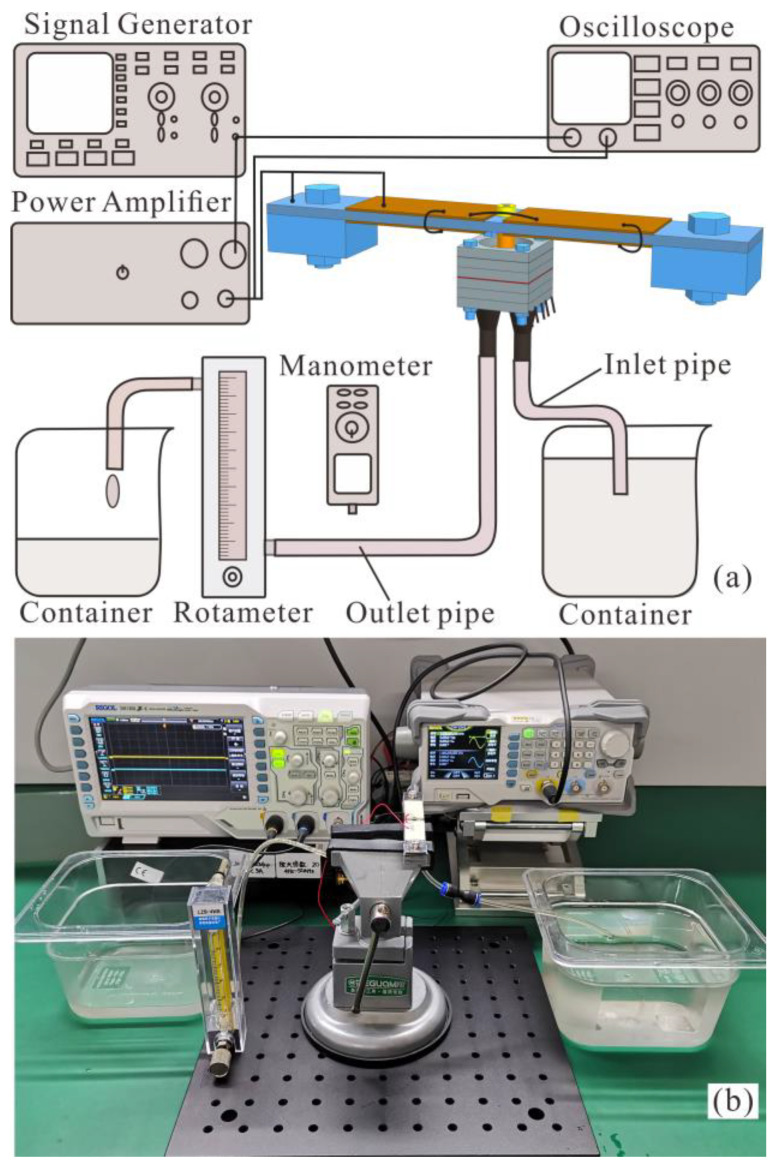
(**a**) Schematic illustration and (**b**) photograph of the experimental setup.

The experimental setup shown in Figure 11 was used to study the characteristics of the designed piezoelectric pump, and tap water was used as the working liquid to carry out all experiments at an average room temperature of about 25 °C. Low voltage sinusoidal signals were generated by a function generator (DG1022, RIGOL, Beijing, China), and amplified by a power amplifier (PA94, Apex Microtechnology, Tucson, AZ, USA) to drive the four piezoelectric sheets simultaneously. The amplitude and frequency of the driving voltage was monitored by a digital oscilloscope (DS1202Z-E, RIGOL, Beijing, China). The flowrate of the piezoelectric pump was measured by a glass rotameter, and the pressure was monitored by a digital manometer. Two types of square gaskets with the weights of 1.8 g and 2.7 g were selected to adjust the weight of the mass blocks.

### 4.2. Results

**Figure 12:**
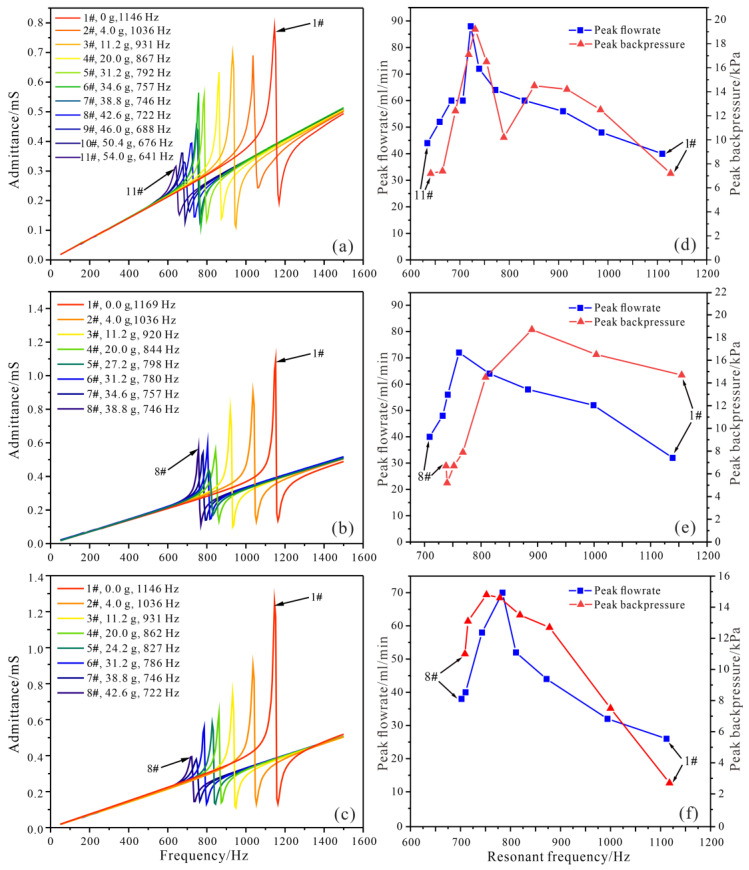
Frequency responses of admittances under different mass blocks of the piezoelectric pump using check valves with (**a**) 5 inlet holes, (**b**) 4 inlet holes, and (**c**) 3 inlet holes; relationships between the peak flowrate and backpressure with resonant frequency of the piezoelectric pump using check valves with (**d**) 5 inlet holes, (**e**) 4 inlet holes, and (**f**) 3 inlet holes.

Firstly, the diaphragm pump using check valves with five inlet holes was experimentally evaluated. The amplitude of the driving voltage was set at 300 V_pp_, the flowrate was measured under zero backpressure, and the backpressure was measured under zero flowrate. Every time the mass blocks were adjusted, the admittance of the piezoelectric pump without liquid was measured by an impedance analyzer (LCR-8201, GW Instek, Taiwan), and the peak admittance frequency was obtained as plotted in Figure 12a. When certain mass blocks were installed, the frequency responses (50~1500 Hz) of the flowrate and backpressure were investigated, respectively, then the peak flowrate and backpressure together with their corresponding resonant frequencies were recorded, as shown in Figure 12d.

The resonant frequency of the piezoelectric pump decreased from 1146 Hz to 641 Hz with the increasing of the weight of the mass blocks from 0 g to 54 g (Figure 12a,d). The peak flowrate and backpressure varied with the resonant frequencies under the different mass blocks, as shown in Figure 12d, and when 38.8 g mass blocks were installed, the maximum of the peak flowrate reached 88 mL/min at 722 Hz, and the maximum of the peak backpressure reached 19.2 kPa at 731 Hz. In this case, the output performance of the piezoelectric pump using the five-inlet-hole valves was optimized by tuning the resonant frequency of the resonator close to the side-curling mode of the valve (744 Hz in Figure 2b).

Next, diaphragm pumps using the check valves with four and three inlet holes were tested, respectively, following the same experimental steps as before. The results for the piezoelectric pump using the four-inlet-hole valves are shown in Figure 12b,e. The adjusting range of the resonant frequency was 746~1169 Hz using mass blocks weighing 0~38.8 g (Figure 12b). The maximum of the peak flowrate reached 72 mL/min at 761 Hz when 27.2 g mass blocks were installed, and the maximum of the peak backpressure reached 18.7 kPa at 890 Hz when 11.2 g mass blocks were installed (Figure 12e). For the piezoelectric pump using the three-inlet-hole valves, the resonant frequency tuning range was 722~1146 Hz with mass blocks weighing 0~42.6 g (Figure 12c). The maximum of the peak flowrate reached 70 mL/min at 789 Hz when 24.2 g mass blocks were installed, and the maximum of the peak backpressure reached 14.8 kPa at 752 Hz when 31.2 g mass blocks were installed (Figure 12f).

A comparison among the simulated side-curling resonant frequency, resonant frequency of the maximum peak flowrate and resonant frequency of the maximum peak backpressure is presented in Table 2. It can be seen that when the resonant frequencies of the piezoelectric pumps using the aforementioned three types of check valves were tuned close to the side-curling resonant frequencies of the check valves, the pumps’ peak flowrates could be maximized, and the deviations between the resonant frequencies were less than 3.04%. Since the phase angle of the valve varied from the antiphase to the inphase (Figure 3) around the side-curling resonant frequency, the actual phase of the valve could be any value between 0° and 180°, and would need to be measured directly to explore the optimal phase in the working mode of the valve in future studies. On the other hand, the resonant frequencies of the maximum peak backpressures deviated from the side-curling resonant frequencies of the valves. Since the backpressure was measured by blocking the outlet (under a zero flowrate), it can be assumed that the working mode of the valve may be changed under different loads.

## 5. Conclusions

In this paper, the optimal operating mode of the bridge-type PDMS valve and a piezoelectric resonator with a tunable resonant frequency were investigated. The analysis indicates that the side-curling mode of the PDMS valve has the advantages of being conducive to liquid flow and having a fast frequency response. To utilize the side-curling mode of the valve, mass blocks of different weights were installed on both ends of a beam-type piezoelectric resonator for tuning its first bending resonant frequency close to the side-curling resonant frequency of the valve. A prototype pump constructed of the piezoelectric resonator and diaphragm pumps using PDMS valves with five, four and three inlet holes was experimentally evaluated. The peak flowrates varied with the resonant frequencies under different mass blocks, and reached their maximum values when the resonant frequencies of the resonator were tuned close to the side-curling mode of the valves. With a driving voltage of 300 V_pp_, the maximum peak flowrates of 88 mL/min, 72 mL/min and 70 mL/min were achieved at 722 Hz, 761 Hz and 789 Hz, respectively, for the diaphragm pumps using five-, four- and -inlet-hole PDMS valves. This study proves that the side-curling mode is the optimal operating mode for the bridge-type PDMS valves, and the output flowrate of a piezoelectric pump can be improved remarkably by tuning the resonant frequency of the resonator to match with the side-curling mode of the check valve.

## Figures and Tables

**Table 1:** Parameters of the piezoelectric resonator and the check valve.

Parameter	Symbol	Value
Elastic beam length	*l*	100 mm
Elastic beam width	*w*	15 mm
Elastic beam thickness	*t*	2.0 mm
Elastic beam density	*ρ*_1_	7850 kg/m^3^
Elastic beam Young’s modulus	*E*_1_	210 GPa
Elastic beam Poisson’s ratio	*μ*_1_	0.288
PDMS density	*ρ*_2_	1030 kg/m^3^
PDMS Young’s modulus	*E*_2_	0.6 MPa
PDMS Poisson’s ratio	*μ*_2_	0.49

**Table 2:** Comparison of optimal operating frequencies between simulation and experiments.

Check Valve Type	Five-Inlet-Hole	Four-Inlet-Hole	Three-Inlet-Hole
Simulated side-curling resonant frequency (Hz)	744	754	791
Resonant frequency of maximum peak flowrate (Hz)	722	761	789
Resonant frequency of maximum peak backpressure (Hz)	731	890	752

## Data Availability

The data presented in this study are available upon reasonable request from the authors.
